# Identification of highly expressed genes and efficient core promoters specific to buffalo skeletal muscles

**DOI:** 10.5194/aab-68-67-2025

**Published:** 2025-01-31

**Authors:** Jieping Huang, Duo Guo, Ruirui Zhu, Haopeng Wang, Chunyan Yang, Deshun Shi, Jianghua Shang

**Affiliations:** 1 Guangxi Key Laboratory of Buffalo Genetics, Reproduction and Breeding, Buffalo Research Institute, Chinese Academy of Agricultural Sciences, Nanning, Guangxi 530001, China; 2 State Key Laboratory for Conservation and Utilization of Subtropical Agro-Bioresources, Guangxi Key Laboratory of Animal Breeding, Disease Control and Prevention, Guangxi University, Nanning, Guangxi 530005, China

## Abstract

An efficient promoter with specific transcriptional activity plays significant roles in the regulation of expression of exogenous genes. The efficient promoter specific to skeletal muscles can achieve high expression of exogenous genes in skeletal muscles. This is of great significance for the targeted improvement of livestock meat quality by combining gene editing and traditional breeding techniques. To identify efficient promoters specific to the skeletal muscles of buffalo, in the present study, a total of 14 genes, *CACNG1*,* LRRC30*,* CACNG6*,* MYOG*,* VGLL2*,* MYOD1*,* KCNA7*,* DUPD1*,* PRR32*,* LBX1*,* IGFN1*,* ACTN3*,* PITX3*, and* MURC*, were firstly screened as skeletal-muscle-specific expressed genes based on high-throughput sequencing data. Among them, only two genes – namely, *VGLL2* and *CACNG1* – were identified to be specifically and efficiently expressed in the skeletal muscles of buffalo by quantitative reverse transcription polymerase chain reaction (RT-qPCR). Then, the transcriptional activity of different truncated fragments of the upstream putative promoter region of* VGLL2* and *CACNG1* were evaluated by the dual luciferase reporter gene detection system in mouse C2C12 cells and buffalo skeletal muscle cells. As a result, both core promoters of *VGLL2* and *CACNG1* were identified to have specifically and efficiently transcriptional activity in skeletal muscle tissue while the transcriptional activity of the core promoters of *VGLL2* was more efficient. These results provide significant information for the targeted improvement of meat quality in buffaloes and other livestock animals.

## 1 Introduction

Buffaloes are abundant and primarily raised for draft power in China and other Asian countries. Due to the increasing agricultural mechanization, the utility of buffaloes in draft power has gradually decreased. Therefore, it is important for the transformation of buffaloes into animals raised for meat production (Naveena and Kiran, 2014). It is well known that intramuscular fat (IMF) content significantly enhances the flavor and juiciness of meat. However, the IMF content is very low in buffaloes, which makes the flavor and taste of buffalo meat inferior to cattle meat (beef) (Huang et al., 2020a). Meanwhile, fat deposition in other depots, such as subcutaneous fat and visceral fat, are considered waste in buffalo production. Breeders desire to specifically increase IMF deposition without affecting fat deposition in other depots (Hudson et al., 2015; Ren et al., 2017). Therefore, it is of great significance to specifically upregulate the key genes of fat deposition in the skeletal muscles in buffaloes.

**Table 1:** Details of primers used for RT-qPCR.

Gene	Primers ( $5^{\prime }\to 3^{\prime }$ )	Length [bp]	Tm [°C]
*CACNG1*	F: AGGCATTTTAACCCAGGCGA	212	60
	R: GACTGCCGCATGACCTCC		
*CACNG6*	F: CACGGGAGAGAATGCACACA	91	60
	R: AACTGCCAAGCCTAGTACCG		
*MYOG*	F: GCAGCGCCATCCAGTACATAG	227	60
	R: CAGATTGTGGGCGTCTGTAGG		
*VGLL2*	F: GCCATGAGCTGTCTGGATGT	249	60
	R: GATGTACTCCGCCTCTGGTG		
*MYOD1*	F: CGCTCCAACTGTTCCGAC	88	60
	R: TGTAGTAAGTGCGGTCGTAGC		
*KCNA7*	F: GTCGTCTCGGTGCTTGTCAT	191	60
	R: AAGAACGGGTCATCGAAGGG		
*DUPD1*	F: CAGTACACCCACGTCAACGA	162	60
	R: CATGTCGCGGTAGTAGTCGG		
*PRR32*	F: CTCTAAGGAAGAAGCACCAGTCA	272	60
	R: GAAGGGCGGTCTCAGCG		
*IGFN1*	F: GATCAATAAGCTGACCGGCG	134	60
	R: CCTTGGGCCTCTTCCGATTC		
*ACTN3*	F: CGCCGAGCAGAGCGGA	204	60
	R: GAGTTGCACCAGGCAGTGAA		
*PITX3*	F: CCTGCTGCGGGACTCACTA	158	60
	R: CCGAATCGCTGTGCTCTTG		
*MURC*	F: AAAACCCGAAAGGTCAGTGCT	245	60
	R: TCCGAAGACAGATCAACTGGG		

There are complex factors that are responsible for the expression of a gene in eukaryote. Among them, the upstream promoter is the most direct regulator in transcriptional regulation (Fraimovitch and Hagai, 2023). Meanwhile, many other regulatory elements, such as transcription factors, regulate the transcription of a gene by binding to promoter (Zabidi and Stark, 2016; Zhang et al., 2022). By binding to the promoter, regulatory elements are typically assembled to form transcriptional switches capable of controlling gene expression (Pandian et al., 2014). Specifically, the core promoter sequence largely determines the expression level of its downstream region (Savina et al., 2023). Interestingly, many genes are expressed at specific times and locations, exerting effects on specific tissues (Kassam et al., 2019; Jiang and Chen, 2022). This is achieved by specific transcriptional regulatory factors acting on the promoter region (Zhao et al., 2022). With the development of gene editing technology, high expression of target genes in specific tissues can be achieved by assembling the tissue-specific promoters with the coding DNA sequence (CDS) region of target genes and inserting them into a safe locus in the genome (Ruan et al., 2015; Gao et al., 2017; Gu et al., 2021). For instance, muscle creatine kinase (MCK) is mainly expressed in muscle tissue; a fragment MCK promoter with PPARG was inserted into the genome to obtain an overexpression of PPARG in skeletal muscles in pigs (Gu et al., 2021). Similarly, myogenin (*MYOG*) is highly expressed in muscles; the promoter of *MYOG* was used to drive the expression of specific genes in muscle tissue in mice (Huang et al., 2012). Therefore, in order to improve the IMF level, the promoter of skeletal-muscle-specific high-expression genes can be used to drive the expression of IMF key genes in skeletal muscle tissue in buffaloes.

To date, several genes, such as MCK and *MYOG*, have been identified to have specific and high expression in skeletal muscles in model animals (Huang et al., 2012; Gu et al., 2021). However, the expression patterns of genes may vary among species. To the best of our knowledge, genes with specific and high expression in skeletal muscles have not been revealed or identified in buffaloes. To identify the efficient core promoters specific to buffalo skeletal muscles, the skeletal-muscle-specific genes are firstly screened, and the core promoters are then identified in buffaloes in the present study. Results of this study provide information significant for the genetic improvement of meat quality in buffaloes.

## 2 Methods

### 2.1 Screening of candidate genes based on RNA sequencing data

The RNA sequencing data of muscle and adipose tissues of buffalo (Huang et al., 2020a) were firstly used to screen the skeletal-muscle-specific highly expressed genes. The parameters used for screening are as follows. The mean fragments per kilobase million (FPKM) value of adipose tissues was lower than 0.3 and that of muscle tissue was higher than 2. The FPKM value of a single adipose tissue was lower than 0.5 and that of a single muscle tissue was higher than 2. Secondly, another RNA sequencing dataset of cattle (Sequence Read Archive, SRA, in the National Center for Biotechnology Information, NCBI, database, PRJNA1000105), including heart, liver, spleen, lung, kidney, muscle, and adipose tissues, was used to further screen the candidate genes obtained based on the buffalo RNA sequencing data. In this step, the mean FPKM value of heart, liver, spleen, lung, kidney, and adipose tissues was lower than 1 and that of muscle tissue was higher than 4.

### 2.2 Sample preparation

The longissimus dorsi muscle, heart, spleen, lung, kidney, and liver tissues were sampled from Binlangjiang buffaloes, including six periods (fetal and 6 months, 1 year, 2 years, 3 years, and 4 years of age), with six individuals for each period. Animals of the fetal and 6-month-old periods were male, and those in other periods were castrated. Other details of the animals were described in a previous study (Huang et al., 2020b). Fetal buffaloes used for the isolation of skeletal muscle cells was obtained from a slaughterhouse (Wufeng United Food Co., Ltd., Nanning, China). All efforts were made to minimize the suffering of the animals. All animal protocols were approved by the Animal Care Committee of the College of Animal Science and Technology, Guangxi University (Gxu-2021-050).

### 2.3 RNA isolation and RT-qPCR analysis

Total RNA was extracted by the TRIzol reagent (Invitrogen, Carlsbad, CA, USA). RNA concentration and quality were measured using a spectrophotometer (Thermo Fisher Scientific) and agarose gel (Biowest). Specific primers used for quantitative reverse transcription polymerase chain reaction (RT-qPCR) were designed by the Primer-BLAST of NCBI (Table 1). Total RNA was reverse transcribed by the PrimeScript RT Reagent Kit with gDNA Eraser (Takara, Dalian, China). qPCR was performed with the SYBR Green I reagent (Takara, Dalian, China). *UXT* and *RPL* were used as reference genes (Feng et al., 2022). The 2^−ΔΔCt^ method was used to calculate the expression level of a candidate gene. Muscle tissue was used as the control group. Three replicates were run per sample.

**Table 2:** Details of primers used for the construction of the pGL3-Basic vector.

Gene	Primers ( $5^{\prime }\to 3^{\prime }$ )	Length [bp]	Tm [°C]
*VGLL2*_1	F: CGG *GGTACC *CCCCAGAAGAGAAACGGATAAA	1954	60
	R: CCG *CTCGAG *GTTTTATCGTCAACTCTCCTCGG		
*VGLL2*_2	F: CGG *GGTACC *GCCCCACAGGTATTTCCGTA	1385	60
	R: CCG *CTCGAG *GTTTTATCGTCAACTCTCCTCGG		
*VGLL2*_3	F: CGG *GGTACC *GAGGACATCCAGGAGAAAGGG	905	60
	R: CCG *CTCGAG *GTTTTATCGTCAACTCTCCTCGG		
*VGLL2*_4	F: CGG *GGTACC *TGGATGCACACGCTCAATTC	581	60
	R: CCG *CTCGAG *GTTTTATCGTCAACTCTCCTCGG		
*CACNG1*_1	F: CGG *GGTACC *CCATGTGCTGTGTGACCGTAG	2210	60
	R: CCG *CTCGAG *CTCGGACCTTCAGGGTTTTG		
*CACNG1*_2	F: CGG *GGTACC *TGCCATCCAGCCATCTCAT	1576	60
	R: CCG *CTCGAG *CTCGGACCTTCAGGGTTTTG		
*CACNG1*_3	F: CGG *GGTACC *TTACCTGCCATTGAAGTGAAGTC	1001	60
	R: CCG *CTCGAG *GGGGTGGAAGCTAAGTTTAGTGT		
*CACNG1*_4	F: CGG *GGTACC *GGTGGAGGGAAGGAAAGAGAA	682	60
	R: CCG *CTCGAG *CCGCTCGAGTTCAGGGTTTTG		

Note that, in the primer sequences, italic letters indicate the restriction sites and underlined letters indicate the protective base of restriction sites.

**Figure 1:**
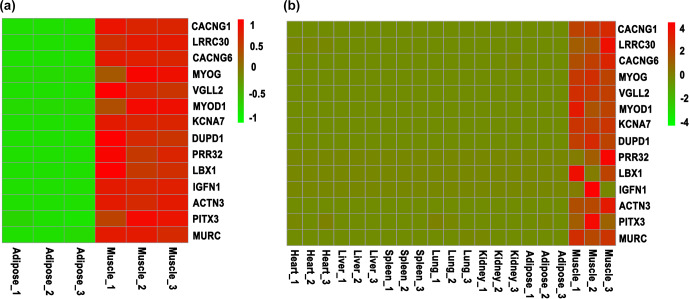
Screening of skeletal-muscle-specific highly expressed genes based on RNA sequencing data of bovine. **(a)** Screening of skeletal-muscle-specific highly expressed genes between muscle and adipose tissue in buffaloes. **(b)** Screening of skeletal-muscle-specific highly expressed genes among heart, liver, spleen, lung, kidney, adipose, and muscle tissue in cattle. Data are presented as the normalization of expression level, which was obtained by the formula (FPKM - mean (FPKM)) / SD.

### 2.4 PCR amplification

Specific primers were designed for the upstream 2000 bp region (putative promoter region) of *VGLL2 *(gene ID: 102390959) and *CACNG1* (gene ID: 102412641) by Primer5.0 (Table 2). Genomic DNA was extracted from the longissimus dorsi muscle of a Binlangjiang buffalo (2 years old) by a Genomic DNA Extraction Kit (Tiangen Biotech, Beijing, China). PCR amplification was performed by a PrimeSTAR GXL DNA Polymerase (Takara, Dalian, China). The PCR production was identified by agarose gel electrophoresis and then stained by nucleic acid dye (Uelandy, Shanghai, China). After staining, the gel was imaged by a gel imaging system.

### 2.5 Vector construction

The PCR production was purified by a Universal DNA Purification and Recovery Kit (Tiangen Biotech, Beijing, China). The recovered fragments and pGL3-Basic plasmids were digested with restriction enzymes *Kpn*I and *Xho*I (Takara, Dalian, China) and then further recovered by a Universal DNA Purification and Recovery Kit (Tiangen Biotech, Beijing, China). Different truncations of the upstream putative promoter regions of *VGLL2* and *CACNG1* were ligated into the pGL3-Basic vector by T4 DNA ligase (Takara, Dalian, China). All the processes were performed according to the manufacturer's instructions. Constructs were verified by direct sequencing.

### 2.6 Cell preparation and culture

The longissimus dorsi muscle of a fetal buffalo was used for the isolation of skeletal muscle cells according to a previous study (Huang et al., 2022). Briefly, fresh longissimus dorsi muscle was digested with 0.2 % collagenase type II (Solarbio, Beijing, China) at 37 ° C until complete digestion. The digested sample was filtered and washed to obtain cells. The cells were cultured with a complete culture medium, Dulbecco's Modified Eagle Medium (DMEM), with 10 % fetal bovine serum and 1 % penicillin–streptomycin, at 37 °C with 5 % CO_2_ for 1 h. Then, the culture medium and non-adherent cells (skeletal muscle cells) were transferred to a new culture dish for further cultivation. When 90 % confluence was reached, the skeletal muscle cells were digested and collected for the following experiments.

The C2C12 cells were purchased from ATCC (Shanghai, China). C2C12 cells were also cultured with a complete culture medium at 37 °C with 5 % CO_2_.

### 2.7 Transfection and dual luciferase analysis

Both buffalo skeletal muscle cells and C2C12 cells were cultured in 48-well plates at 37 °C with 5 % CO_2_. When cells reached 70 % confluence, plasmid transfection was performed using Lipofectamine 3000 (Invitrogen, Carlsbad, CA, USA) according to the manufacturer's instructions. The pGL3-Basic empty vector and pRL-TK plasmid were co-transfected at a ratio of 10:1 as control. Each truncation of the promoter of CACNG1 or VGLL2 that ligated into the pGL3-Basic plasmid was co-transfected with pRL-TK plasmid with the 10:1 ratio as well. Other details can be found in a previous study (Huang et al., 2016). Cells were collected 48 h after the transfection. Luciferase activity was detected on a microplate reader according to the Dual-Luciferase Reporter Assay System operation instructions (Invitrogen, Carlsbad, CA, USA) (Huang et al., 2016).

**Figure 2:**
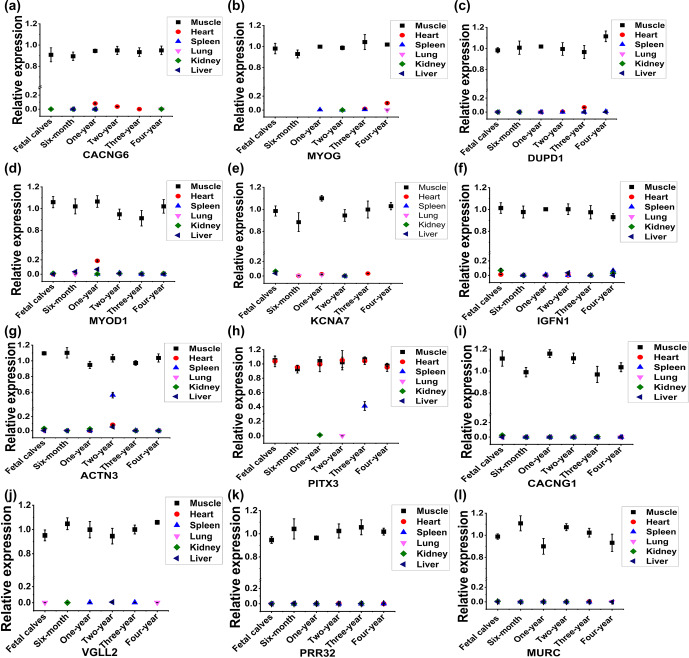
Identification of skeletal-muscle-specific highly expressed genes by RT-qPCR in buffaloes. Expression profile of *CACNG6* **(a)**, *MYOG* **(b)**, *DUPD1* **(c)**, *MYOD1 * **(d)**, *KCNA7* **(e)**, *IGFN1* **(f)**, *ACTN3* **(g)**, *PITX3* **(h)**, *CACNG1* **(i)**, *VGLL2* **(j)**, *PRR32* **(k)**, and *MURC* **(l)** across six organs (heart, muscle, spleen, lung, kidney, and liver) and six development stages (fetal and 6 months, 1 year, 2 years, 3 years, and 4 years of age) in buffaloes. Data are presented as mean ± SD.

### 2.8 Statistical analysis

Data were compared and analyzed by SPSS Statistics software using ANOVA with a post hoc comparison (Tukey test). Data were presented as means ± SD by GraphPad Prism 7 software. A p<0.05 was considered to indicate a statistically significant difference.

## 3 Results

### 3.1 Screening of skeletal-muscle-specific highly expressed genes based on RNA sequencing data

To screen the skeletal-muscle-specific highly expressed genes, RNA sequencing data of muscle and adipose tissues of buffalo (Huang et al., 2020a) were firstly used. As shown in Table S1 in the Supplement, a total of 72 genes were highly expressed in muscle tissue, whereas they were almost not expressed in adipose tissue. Meanwhile, another RNA sequencing dataset, including heart, liver, spleen, lung, kidney, adipose, and muscle tissue of cattle, was downloaded and used to identify the specific highly expressed genes. At last, only 14 candidate genes were obtained (Table S2), though *LBX1* and *IGFN1* show variable expression in the muscle tissue of cattle (Fig. 1). Thus, 14 candidate genes (*CACNG1*,* LRRC30*,* CACNG6*,* MYOG*,* VGLL2*,* MYOD1*,* KCNA7*,* DUPD1*,* PRR32*,* LBX1*,* IGFN1*,* ACTN3*,* PITX3*, and* MURC*) were finally selected for the following validation by RT-qPCR.

**Table 3:** Sequence of the core promoter of VGLL2 and CACNG1.

Gene	Sequence of the core promoter
*VGLL2*	CGAGCGCAGAGACTCTCCTTCACCTCGTCACCCTGGATGCACACGCTCAATTCTCCACTGTGAGGTCGGGGCTCTCCCCAAAGACCCTTCTCCACCACGCCACAACTTCCTCTCTCCAAATGTAAGATCCCCGGTTCCTCCCTCTCCTCTCCGTCCTCCTGTCCCTCCGGCGTTAGCCTGGAAAATCCCTAGTCCTCAACGGTGCGAAGTCTCAGCGCTCAAATTCCGGGCTGCGCAGGTCGCATAGGCTCGCCGCCGGGTGTCCAGGGAAAACGCACCGGCAGCCCCTCGGTCCCTAGTCCCTCTCACTCCATTGGCCCCCCGGTCCCCGCCCCCGACGCCCCCCTCCCCCCGCCTCTGCGGCTCAGGAGTGGCGTCAGCACGCCTGCCCCGCGTGGG
*CACNG1*	GGTGGAGGGAAGGAAAGAGAACTGACAAGGAAATAGTGCAGGAGCGACCTGGGGTGGCTTGTGGGGGTTCAGGTCTAGAGCGTGCTCCCCTGGGCCGTGGGCGCCAGAGGCCCCGGGTGGGGCCCCCCAGGCCCCAGAACAGAGTCCCATTCACCTGGTTCCAGGCGAGGACAGAGGCCTGTGTCCCTGGGTCTGCACCCTCCCCCAGCTCACTCCTCTTTCAGCCCCAGCAGGAAGAGGCTGGCACGCGTAGCGCCAGGCCTGTGCCCCTCGCCCTCCCGGCTCAGGCCCCCTGAGCCGGGCTCTGAGGGTGGCGAGTCTGCAGCCCGTCTGAGCCTTCTCTCCTGCCCCACCCTGGTGTGCTCCTGGCCCCCGGGAGGGGAGCCAGCCCTGAAACCCAAGGAGCAGCCGTCCTCGGTGAGGCTCCGGGGTGTGCATTAATAGAAGCTTCCTCAGGCCTCTGACAC

**Figure 3:**
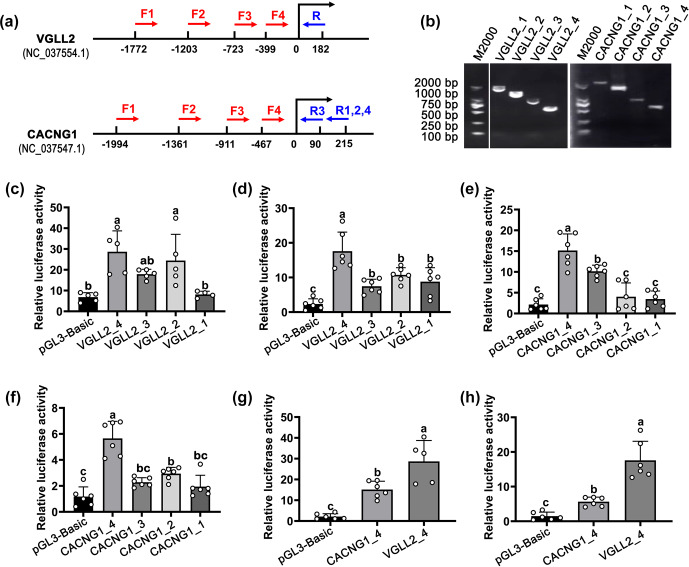
The identification of the core promoter of *CACNG1* and *VGLL2*. **(a)** The position of primers in *CACNG1* and *VGLL2*. For *VGLL2*, the upstream primers, F1, F2, F3, and F4, shared the downstream primer R. For *CACNG1*, the upstream primers, F1, F2, and F4, shared the downstream primer R1,2,4; F3 and R3 are a pair of primers. **(b)** Detection of the PCR products of fragments with different lengths of the putative promoter is performed by agarose gel electrophoresis. **(c)** The relative luciferase activity of the four fragments with different lengths of the putative promoter of *VGLL2* in C2C12 cells. **(d)** The relative luciferase activity of the four fragments with different lengths of the putative promoter of *VGLL2* in buffalo skeletal muscle cells. **(e)** The relative luciferase activity of the four fragments with different lengths of the putative promoter of *CACNG1* in C2C12 cells. **(f)** The relative luciferase activity of the four fragments with different lengths of the putative promoter of CACNG1 in buffalo skeletal muscle cells. **(g)** Comparison of the relative luciferase activity between *VGLL2*_4 and *CACNG1*_4 fragments. Data are presented as mean ± SD. The lowercase letters indicate a p<0.05 .

### 3.2 Expression profile analysis of the candidate genes by RT-qPCR

To analyze the expression profile of the 14 candidate genes by RT-qPCR, specific primers were designed and validated by PCR agarose gel electrophoresis. As shown in Fig. S1a in the Supplement, PCR production of *LBX1* showed non-specific amplification, and no fragment was detected by the primers of *LRRC30*. Thus, a total of 12 pairs of primers of candidate genes was available, including *CACNG6*,* MYOG*,* DUPD1*,* MYOD1*,* KCNA7*,* IGFN1*,* ACTN3*,* PITX3*, *CACNG1*,* VGLL2*,* PRR32*, and* MURC* genes (Fig. S1a in the Supplement). Further, the expression profile of the 12 candidate genes was analyzed across six organs (heart, muscle, spleen, lung, kidney, and liver) and six development stages (fetal and 6 months, 1 year, 2 years, 3 years, and 4 years of age) in buffaloes (Fig. 2). Compared to muscle tissue, some candidate genes had low expression in the heart at a specific stage, such as *CACNG6* at the 1-year-old stage, *MYOG* at the 4-year-old stage, *DUPD1 *and *KCNA7* at the 3-year old stage, and *MYOD1* at the 1-year-old stage (Fig. 2a–e). Similarly, compared to muscle tissue, *IGFN1*, *ACTN3*, and *PITX3* have low expression in the spleen at 4-year-old, 2-year-old, and 3-year-old stages, respectively (Fig. 2f–h). Meanwhile, *PITX3* had a relatively high expression in the heart at the 3-year-old stage compared to that in muscle tissue (Fig. 2h). Thus, four genes – namely, *CACNG1*, *VGLL2*, *PRR32*, and *MURC* – showed specific and relatively high expression in the muscle tissue in buffaloes (Fig. 2i–l).

To obtain a more reliable result, we further checked the expression profile of the four genes in rats or humans based on the available data in NCBI. Among them, *CACNG1* and* VGLL2* had specific and high expression in muscles, while *PRR32* and *MURC* showed considerable expression levels in muscles and other tissues in rats or humans (Fig. S1b–e). At last, *CACNG1* and *VGLL2* were selected for the following identification of promoter activity.

### 3.3 Identification of the transcriptional activity of *CACNG1* and *VGLL2* promoter

To identify the core promoter, transcriptional activity of the upstream ∼2000 bp region of the candidate gene was detected by the dual-luciferase transcription reporter system. For each candidate gene, four fragments with different lengths of the putative promoter were cloned and ligated to the pGL3-Basic vector (Fig. 3a and b). The pGL3-Basic vector carrying putative promoter was co-transfected with the pRL-TK vector into mouse C2C12 cells and buffalo myocytes, respectively. For the *VGLL2* gene, the *VGLL2*_4 fragment showed the highest activity in both kinds of cells (Fig. 3a and d; p<0.05 ). Similarly, among the fragments of the *CACNG1* gene, *CACNG1*_4 showed the highest activity in both C2C12 cells and buffalo myocytes (Fig. 3e and f; p<0.05 ). Then, the transcriptional activity of *VGLL2*_4 and*CACNG1*_4 fragments were further compared. As shown in Fig. 3g and h, the *VGLL2*_4 fragment had a higher efficiency than the *CACNG1*_4 fragment ( p<0.05 ). The *VGLL2*_4 and the *CACNG1*_4 fragments possess a 399 and 467 bp promoter subsequence, respectively (Table 3). Thus, both of them are with specifically and efficiently transcriptional activity in skeletal muscle tissue, while that of *VGLL2* is more efficient. These two core promoters can be used for overexpression of a target gene in the muscle of buffalo by transgenic or gene editing technology.

## 4 Discussion

In mammals, every individual cell possesses the same genetic material. However, gene expression profiles are different between tissues and organs, allowing them to play special roles in special anatomical position and development stages. Although the expression of a gene is generally regulated by a complex gene network, this is mainly achieved by affecting the transcriptional activity of the promoter. Thus, the promoter is the key hub for the expression of a gene. In the present study, skeletal-muscle-specific expression genes were screened and identified; the core promoter with specifically high efficiency in skeletal muscles, which can be used for the targeted regulation of muscle development and genetic improvement of meat quality in buffaloes, was revealed.

Muscle development and meat quality are important economic traits for livestock raised for meat production. Skeletal-muscle-specific expression genes play significant roles in the regulation of muscle development and meat quality. The myogenic regulatory factors (*MYOG*, *MYOD1*, *MRF4*, and *MYF5*) (Hernández-Hernández et al., 2017; Zammit, 2017) and *MYHC* (Schiaffino and Reggiani, 2011) are highly expressed in muscle tissue and regulate the development of muscle by controlling the expression of many key genes. Among them, only *MYOG* and *MYOD1* showed a high expression level in muscle tissue in buffaloes based on our RNA sequencing data (Fig. 1). By RT-qPCR identification, *MYOG* had a considerable expression level in the heart of 4-year-old buffaloes (Fig. 2b), and *MYOD1 *was identified in the heart and the liver of 12-month-old buffaloes (Fig. 2g). Thus, the myogenic regulatory factors are not the skeletal-muscle-specific expressed genes in buffaloes. Among the other 10 candidate genes, six genes – namely, *CACNG6*, *KCNA7*, *DUPD1*, *IGFN1*, *ACTN3*, and *PITX3* – can be detected in the heart or spleen based on RT-qPCR detection (Fig. 2a, c, e–h). Only four genes – namely, *CACNG1*, *VGLL2*, *PRR32*, and *MURC* – were detected mainly in skeletal muscle tissue by RT-qPCR detection (Fig. 2i–l). However, based on the data in NCBI, *PRR32* and *MURC* show considerable expression levels in muscles and other tissues in rats or humans (Fig. S1d and e). At last, only *VGLL2* and *CACNG1* were considered to have high and specific expression in the skeletal tissue in buffaloes. *VGLL2* has been well studied and proven to promote the differentiation in skeletal muscles in mammals (Maeda et al., 2002; Chen et al., 2004; Honda et al., 2017). *CACNG1* is a significant auxiliary of a voltage-gated calcium channel (Jiang et al., 2024) and plays a role in the atrophy of myotubes (Okada et al., 2021) and hypokalemic periodic paralysis (Li et al., 2005). However, research on the specific role of *CACNG1* in skeletal muscles is very limited.

A gene can be expressed in specific tissues primarily due to the combined action of upstream promoter sequences and specific transcription factors expressed in specific tissues (Zabidi and Stark, 2016; Mantovani et al., 2021; Zhang et al., 2022). Thus, in specific tissues, the specific promoter is crucial for a gene expression. Previously, several promoters, such as MCK and *MYOG* promoters, were considered to be skeletal-muscle-specific (Huang et al., 2012; Gu et al., 2021). However, these two genes are not specifically expressed in the skeletal muscles in buffaloes. In this study, both *VGLL2* and *CACNG1* have a specifically high expression level in the skeletal muscles in buffaloes. Specifically, the significant roles of *VGLL2* in skeletal muscles have been widely studied in mammals (Maeda et al., 2002; Chen et al., 2004; Honda et al., 2017). However, to the best of our knowledge, the promoter of *VGLL2* has not been used to drive the expression of exogenous genes in skeletal muscle tissue to specifically enhance IMF deposition in muscle tissue. In the present study, the core promoters of *VGLL2* and *CACNG1* have been identified, and the core promoter of *VGLL2* has a higher transcriptional activity than that of *CACNG1*. Therefore, both of them can be used as skeletal-muscle-specific promoters, and the core promoter of *VGLL2* is superior in efficiency.

## 5 Conclusions

The present study reveals that *VGLL2* and *CACNG1* are specifically and highly expressed in the skeletal muscle tissue of buffaloes. Both core promoters of *VGLL2* and *CACNG1* show skeletal-muscle-specific and efficient transcriptional activity, while the former is superior in efficiency. These results provide an efficient promoter that can be used to specifically enhance IMF deposition in the skeletal muscles, which is significant for the improvement of meat quality in buffaloes.

## Supplement

10.5194/aab-68-67-2025-supplement The supplement related to this article is available online at: https://doi.org/10.5194/aab-68-67-2025-supplement.

## Data Availability

The original data are available upon request from the corresponding author.
