# Supplementary material for: Identification of highly expressed genes and efficient core promoters specific to buffalo skeletal muscles
Source: Arch Anim Breed. 2025 Jan 31;68(1):67–76. doi: 10.5194/aab-68-67-2025 (PMC13228153; doi:10.5194/aab-68-67-2025)
Supplement: The supplement related to this article is available online at: https://doi.org/10.5194/aab-68-67-2025-supplement. [file aab-68-67-2025-supplement.zip › aab-68-67-2025-supplement-title-page.pdf]

Supplement of Arch. Anim. Breed., 68, 67–76, 2025
https://doi.org/10.5194/aab-68-67-2025-supplement
© Author(s) 2025. CC BY 4.0 License.

Open Access
Archives Animal Breeding

Supplement of

Identification of highly expressed genes and efficient core promoters specific to buffalo skeletal muscles

Jieping Huang et al.

Correspondence to: Jieping Huang (huangjieping1989@126.com) and Jianghua Shang (shang@163.com)

- aab-68-67-2025-supplement-title-page.pdf
- Figure S1-S2.pdf
- Table S1.xlsx

The copyright of individual parts of the supplement might differ from the article licence.
