# Supplementary material for: Identification of highly expressed genes and efficient core promoters specific to buffalo skeletal muscles
Source: Arch Anim Breed. 2025 Jan 31;68(1):67–76. doi: 10.5194/aab-68-67-2025 (PMC13228153; doi:10.5194/aab-68-67-2025)
Supplement: The supplement related to this article is available online at: https://doi.org/10.5194/aab-68-67-2025-supplement. [file aab-68-67-2025-supplement.zip › Figure S1-S2.pdf]

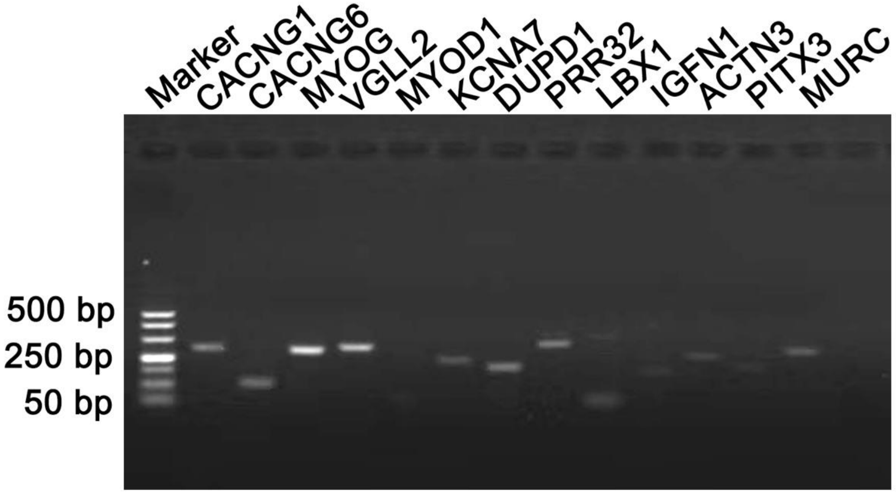

Figure S1 Detection of the PCR production of the candidate genes by agarose gel electrophoresis.

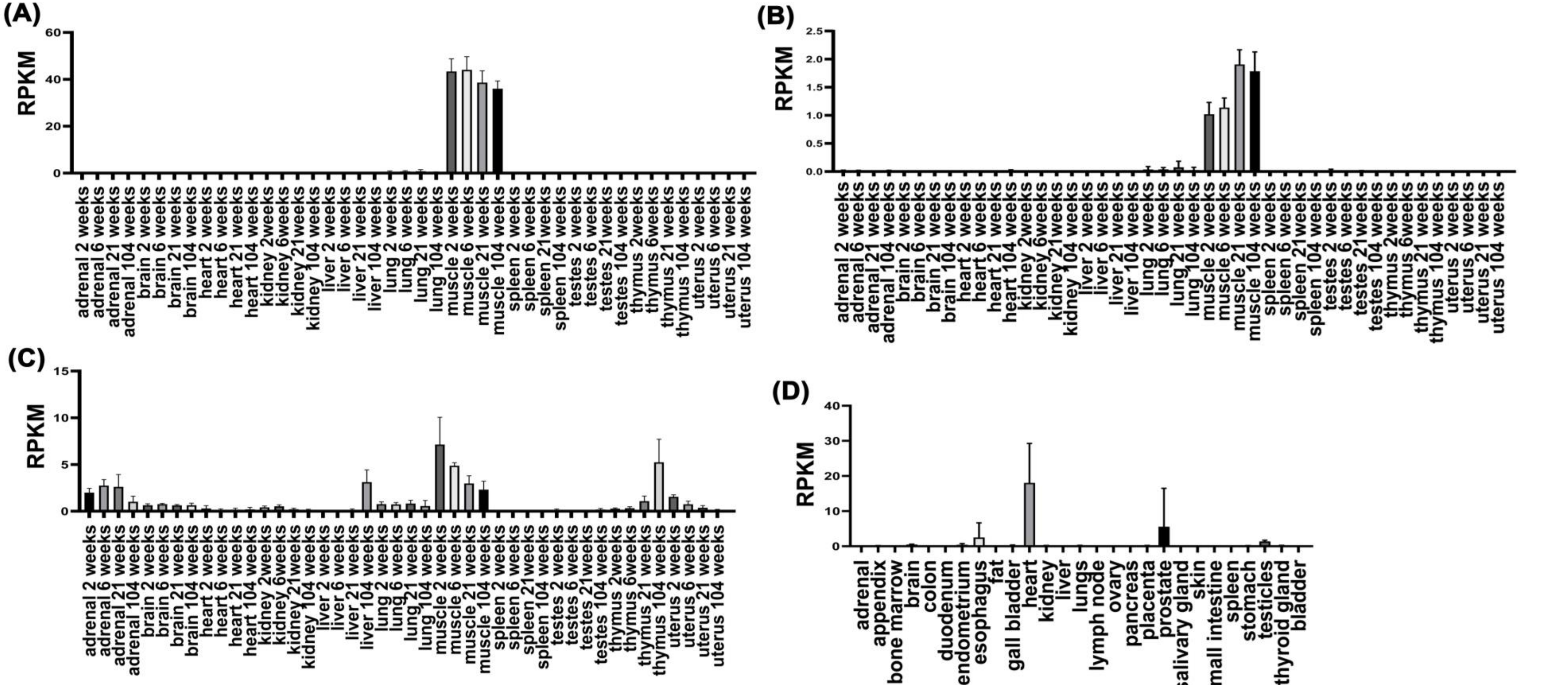

Figure S2. Expression profile of four candidate genes based on the available data in NCBI. (A) Expression profile of CACNG1 in rats based on the data in NCBI (PRJNA238328). (B) Expression profile of VGLL2 in rats based on the data in NCBI (PRJNA238328). (C) Expression profile of PRR32 in rats based on the data in NCBI (PRJNA238328). (D) Expression profile of MURC in humans based on the data in NCBI (PRJEB4337).
